# Comparison of Immunosuppressive and Angiogenic Properties of Human Amnion-Derived Mesenchymal Stem Cells between 2D and 3D Culture Systems

**DOI:** 10.1155/2019/7486279

**Published:** 2019-02-18

**Authors:** Vitale Miceli, Mariangela Pampalone, Serena Vella, Anna Paola Carreca, Giandomenico Amico, Pier Giulio Conaldi

**Affiliations:** ^1^Department of Laboratory Medicine and Advanced Biotechnologies, IRCCS-ISMETT (Istituto Mediterraneo per i Trapianti e Terapie ad Alta Specializzazione), Palermo, Italy; ^2^Ri.MED Foundation, Palermo, Italy; ^3^Anemocyte S.r.l., Gerenzano, Italy; ^4^Department of Research, IRCCS-ISMETT, Palermo, Italy

## Abstract

The secretion of potential therapeutic factors by mesenchymal stem cells (MSCs) has aroused much interest given the benefits that it can bring in the field of regenerative medicine. Indeed, the *in vitro* multipotency of these cells and the secretive capacity of both angiogenic and immunomodulatory factors suggest a role in tissue repair and regeneration. However, during culture, MSCs rapidly lose the expression of key transcription factors associated with multipotency and self-renewal, as well as the ability to produce functional paracrine factors. In our study, we show that a three-dimensional (3D) culture method is effective to induce MSC spheroid formation, to maintain the multipotency and to improve the paracrine activity of a specific population of human amnion-derived MSCs (hAMSCs). The regenerative potential of both 3D culture-derived conditioned medium (3D CM) and their exosomes (EXO) was assessed against 2D culture products. In particular, tubulogenesis assays revealed increased capillary maturation in the presence of 3D CM compared with both 2D CM and 2D EXO. Furthermore, 3D CM had a greater effect on inhibition of PBMC proliferation than both 2D CM and 2D EXO. To support this data, hAMSC spheroids kept in our 3D culture system remained viable and multipotent and secreted considerable amounts of both angiogenic and immunosuppressive factors, which were detected at lower levels in 2D cultures. This work reveals the placenta as an important source of MSCs that can be used for eventual clinical applications as cell-free therapies.

## 1. Introduction

Adult stem cells are extensively used for regenerative medicine because of their multilineage potential and regenerative properties. These cells exist in different tissues, including fat [1], bone marrow [2], the umbilical cord [3], and placenta tissue [4], where they participate in the maintenance of stem cell niches and tissue homoeostasis [5]. Though the pathophysiologic functions of mesenchymal stem cells (MSCs) are under investigation, the *in vitro* multipotency of these cells suggests a role in tissue regeneration, wound healing, and/or tissue repair after transplantation [6]. Indeed, MSCs are capable of self-renewal and differentiation into several mesenchymal lineages both *in vitro* and *in vivo*, including fat, bone, and cartilage [7]. In addition, several studies have shown that MSCs undergo differentiation into the osteogenic phenotypes when cultured in the presence of specific supplements and these cells were utilized as a source for osteogenic tissue regeneration [8]. Across the last decade, MSCs have also been proven to have both immunomodulatory and angiogenic properties that suppress proliferation/activation of immune cells [9] and promote wound healing *in vivo* [10]. Despite the availability of various cell sources for the use of MSCs in the field of regenerative medicine, the ethical issues regarding the source have become an important clinical concern. Indeed, most of the data on this topic have been thus far generated using bone marrow-derived MSCs (BM-MSCs) [11], while increasing evidence supports the use of neonatal tissues, such as umbilical cord tissue and placenta tissue (e.g., amniotic membrane) [12, 13], as better sources of MSCs. Placenta-derived MSCs (PD-MSCs) have several advantages, such as being abundant, easy to obtain without invasiveness, and readily cultured to a sufficient number for transplantation, thus precluding ethical issues concerning allografting [14]. Furthermore, placenta tissue derives from pregastrulation embryonic cells, conferring its plasticity to the derived cells [14]. Recently, the therapeutic effect of PD-MSCs in the field of regenerative medicine has been shown [15]. Indeed, different types of placenta cells have been described [4], and among these, human amnion-derived mesenchymal stem cells (hAMSCs) have been shown to have immunosuppressive properties both *in vitro* and *in vivo* [16, 17]. Tuca et al. found that hAMSCs participated in both angiogenesis and reepithelialization *in vivo* [18] and the beneficial effect of hAMSCs in inhibition of inflammation and induction of neuronal repair in autoimmune encephalomyelitis mice has been shown [17].

Notably, it has been demonstrated that the main mechanism for MSCs' beneficial effects on injured tissue is represented by their capacity to migrate into damaged areas and exert a trophic effect because of secretion of bioactive factors acting on the injured microenvironment to facilitate tissue repair. On the other hand, another hypothetical mechanism refers to the differentiation of MSCs into functional cells that replace damaged tissue. However, there is evidence concerning poor grafting of transplanted MSCs in spite of substantial therapeutic effects in lung and kidney cartilage injuries, diabetes, myocardial infarction, and other diseases. Tissue repair mechanisms through transplantation of MSCs are most likely due to the production of cytokines and paracrine factors, though this is currently a subject of some debate [19, 20]. An *in vitro* study showed that the conditioned medium produced by umbilical cord MSCs promotes cutaneous wound healing [3], and various studies indicate that amnion-derived cells secrete soluble factors with immunomodulatory capacity [13]. It has also been shown that the *in vivo* administration of conditioned medium derived from hAMSCs favored the repair process after acute myocardial infarction in mouse models [21] and was able to reduce lung fibrosis in a bleomycin mouse model [22]. Moreover, prostaglandin-mediated immunosuppressive effects were shown for conditioned medium derived from hAMSCs [23]. In recent years, microvesicles extracted from supernatant of MSC cells have been used to induce angiogenesis *in vitro* and to treat both kidney injury and myocardial damage in mouse models [24–27]. Therefore, MSC-derived extracellular vesicles such as exosomes (EXO) may contribute to the outcomes of MSC-based therapies [28]. Recently, EXO received attention due largely to a study on a severe graft versus host disease (GVHD) treated with MSC-derived EXO, suggesting that the effect of MSCs could be also reproduced, at least in part, by MSC-derived EXO treatment [29]. Recent data have shown a remarkable paracrine potential of extracellular vesicles derived from human amniotic stem cells [30], and Bier et al. found that the treatment of Duchenne patients' myoblasts with exosomes secreted by hAMSCs increased the differentiation of these cells and decreased the expression of fibrogenic genes [31]. In addition, a mechanism of immunosuppression has been observed for exosomes secreted by human placental explants *in vitro* [32].

In this scenario, MSC-based therapeutics is a promising approach in the field of regenerative medicine, though the beneficial effects in initial small-scale clinical studies are often not confirmed by large clinical trials, indicating the urgent need for further optimization of cell-based therapy [33, 34]. There are different approaches to improving the efficacy of MSC-based therapeutics, and MSC preparation as spheroids represents one method of optimization. Previous studies have shown that culturing MSCs as three-dimensional (3D) aggregates is a simple and reproducible method, which can avoid the disadvantages associated with MSC culture as a monolayer [35]. MSCs are commonly cultured as a two-dimensional (2D) monolayer using conventional tissue culture techniques. These 2D systems inadequately reproduce the *in vivo* microenvironment of stem cells, and this has a profound influence on their biological functions, including their replicative ability and their differentiation capabilities [36]. To resolve these problems and mimic, as far as possible, the *in vivo* microenvironment, several 3D culture systems have been developed and spheroid clusters of cells formed by self-assembly represent one of the best models for the 3D culture [36–39]. 3D cell spheroids prevent cell apoptosis and promote cell stabilization after engraftment in ischemic tissue [40]. Cells within the spheroid are naturally exposed to hypoxia, which naturally preconditions an ischemic environment. Recently, it has been shown that Wharton jelly MSCs seeded on decellularized amniotic membrane scaffolds proved to have higher wound healing capabilities when transplanted onto skin injuries of an SCID mouse model than MSCs alone, showing that a 3D environment can prime MSCs to a more therapy-driven phenotype [10]. Self-assembly into spheroid-like structures enables greater cell-cell and cell-matrix interactions [41], and this natural self-aggregation is an effective system for priming these cells towards a paracrine activity that would further promote therapeutic potential [38]. In contrast to monolayer culture, culturing MSCs as 3D aggregates causes substantial changes in the pattern of gene expression [39]. Several studies have shown that MSCs cultured in 3D spheroid conditions exhibited an enhanced anti-inflammatory effect [37] and secreted higher levels of different cytokines with respect to 2D cultures [42]. Even *in vivo*, 3D MSCs seem to exhibit increased therapeutic potential for myocardial ischemia [43] and critical limb ischemia [44]. Thus, these findings identify aggregation of MSCs as a procedure to enhance their therapeutic potential, including anti-inflammation and angiogenesis to tissue injury sites.

The aims of this study were to explore the placenta tissue as a new source of MSCs with regenerative therapeutic properties and to test the hypothesis that the natural self-aggregation of these cells is an effective system for priming MSCs towards a paracrine activity that would further promote tissue regeneration, avoiding the use of cells in the final medicinal product.

## 2. Materials and Methods

### 2.1. Cell Culture

Human umbilical vein endothelial cells (HUVECs), human bone marrow mesenchymal cells (BM-MSCs), and human dermal fibroblast (HDFa) were obtained from ATCC (USA). HUVECs were maintained in endothelial cell basal medium (Clonetics, MD) supplemented with BulletKit (EBM-2) (Lonza, CH) on a culture flask coated with 0.1% gelatin and maintained at 37°C with 5% CO_2_. BM-MSCs and HDFa cells were grown in DMEM medium (Gibco Invitrogen, USA) supplemented with 10% fetal bovine serum (FBS) (HyClone, USA), 2 mmol/l L-glutamine, 100 U/ml penicillin, and 100 *μ*g/ml streptomycin (Gibco Invitrogen, USA) at 37°C with 5% CO_2_.

### 2.2. Isolation and Culture of Human Amnion Mesenchymal Stem Cells

hAMSCs were isolated from amnion of human term placenta (38–40 weeks of gestation) of healthy donors within 6 hours of birth, using a previously described protocol [23]. Written informed consent and the procedure were approved by ISMETT's Institutional Research Review Board (IRRB). Informed consent was obtained from each donor. The amnion was manually separated from the chorion and washed several times in phosphate-buffered saline (PBS). It was then cut into small pieces of 3 × 3 cm^2^, and each fragment was decontaminated with a brief incubation in PBS with 2.5% Esojod (Esoform, Italy) for 3 minutes in PBS containing 500 U/ml penicillin, 500 mg/ml streptomycin, 12.5 mg/ml amphotericin B, and 1.87 mg/ml cefamezin (Pfizer, Italy) and 5 minutes in PBS containing 100 U/ml penicillin and 100 mg/ml streptomycin. Decontaminated fragments were incubated for 9 minutes at 37°C in HBSS (Lonza, CH) containing 2.5 U/ml dispase (Corning, NY, USA). The fragments were then incubated for 5 minutes at room temperature in complete RPMI 1640 medium (Invitrogen, USA) supplemented with 10% fetal bovine serum (FBS) (HyClone, USA) and subsequently digested with 0.94 mg/ml collagenase A (Roche, Germany) and 20 mg/ml DNase (Roche, Germany) for 2.5 hours at 37°C. The digest was subsequently filtered with both 100 *μ*m and 70 *μ*m cell strainers (BD Falcon, USA), pelleted by centrifugation at 150 − 300 g for 10 minutes and resuspended in a complete RPMI 1640 medium (Invitrogen, USA) supplemented with 10% fetal bovine serum (FBS) (HyClone, USA) for cell counting. Harvested cells were cultured in polystyrene culture dishes (Corning, NY, USA) at 37°C with an atmosphere of 5% CO_2_ in Chang Medium (Irvine Scientific, USA) for the first passage. To obtain hAMSCs at different passages, isolated cells were plated at a density of 1 × 10^4^/cm^2^, and after reaching the confluence, adherent cells were trypsinized and then subcultured until passages 3-5.

### 2.3. Mesenchymal Stem Cell Spheroid Formation

The cells at the second passage were cultured in 2 ml of culture medium (5 × 10^5^ cells/ml) in a suspended state (3D) in 6-well ultralow attachment plate (Corning, NY, USA), which facilitates spheroid formations and their maintenance. hAMSC spheroid cultures were grown in DMEM serum-free medium at 5% CO_2_, at 37°C. In order to perform cell morphology studies, analysis of gene expression, and cell differentiation studies, we obtained single cells after 3 days of spheroid formation by incubation with 0.25% trypsin/EDTA for 5–10 minutes (depending on the size of spheroids) with gentle pipetting every 2–3 minutes.

### 2.4. Flow Cytometric Phenotypic Analysis

Cells were harvested and washed twice with FACS buffer (PBS containing 0.3% BSA and 0.1% NaN_3_). Cells were incubated with diverse antibodies against each cell surface antigen, such as CD90, CD73, CD13, CD45, and HLA-DR (BD Biosciences, USA) on ice for 30 minutes. Cells were then washed twice with FACS buffer and analyzed using the FACS Aria II flow cytometer and FACS Diva software version 6.1.2 (BD Biosciences, Sunnyvale, CA, USA).

### 2.5. Conditioned Media Preparation

For CM collection from 2D culture, the cells at the second passage were plated in a 100 × 17 mm dish (Nunc, Wiesbaden, Germany) at 5 × 10^5^ cells/ml in 10 ml of complete DMEM medium with 10% FBS for 2 days until 90% confluence. The medium was then replaced with serum-free DMEM medium, and the cells were grown for 3 days. For CM collection from 3D culture, the cells were grown as described above. After 1 day of culture, we observed initial spheroid formation and the medium was changed and, after 3 days of conditioning, was collected. The supernatant from both culture systems was centrifuged, filtered using a 0.2 *μ*m sterile filter, and frozen at -80°C until use. Supernatants were used as exosome-depleted medium conditioned by hAMSCs grown as a monolayer (2D CM-exo), complete medium conditioned by hAMSCs grown as a monolayer (2D CM), exosome-depleted medium conditioned by hAMSCs grown as spheroids (3D CM-exo), and complete medium conditioned by hAMSCs grown as spheroids (3D CM).

### 2.6. Isolation of Exosomes

**Figure 1:**
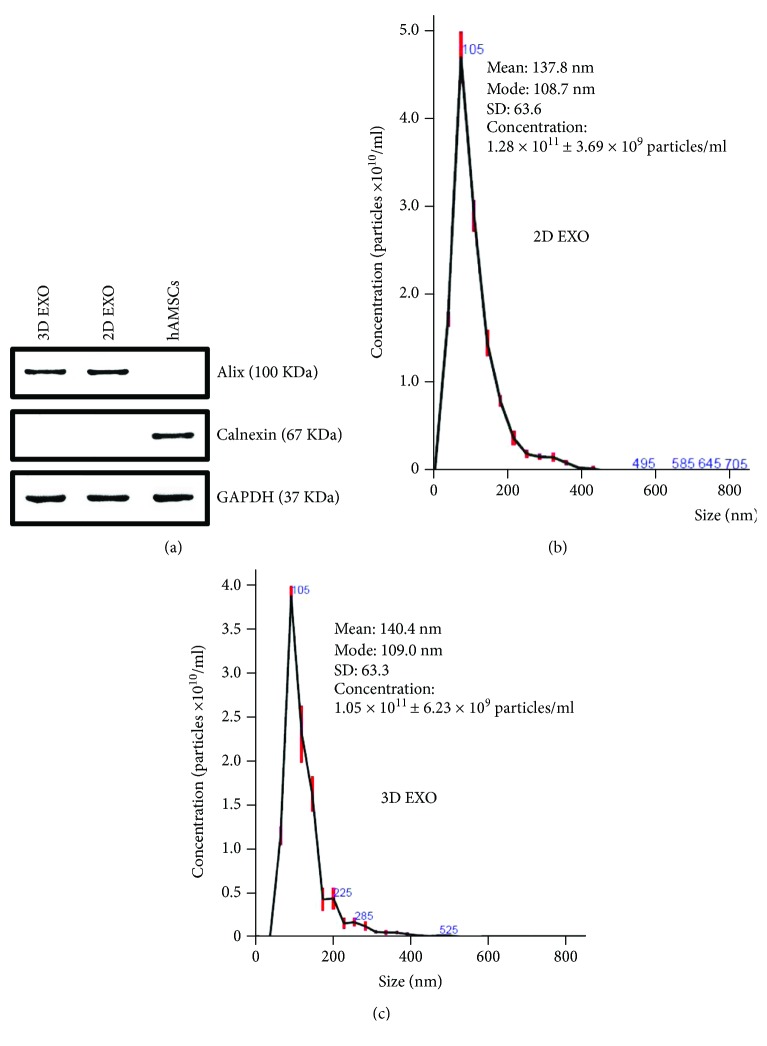
Characterization of exosomes secreted by hAMSCs grown as both monolayer and spheroids. (a) Western blot analysis of extracts prepared from hAMSCs and exosomes isolated from hAMSCs cultured as both monolayer and spheroids. (b) Size of exosomes isolated from hAMSCs cultured as monolayer (2D cultures). (c) Size of exosomes isolated from hAMSCs cultured as spheroids (3D cultures). Exosomes derived from 2D cultures (2D EXO). Exosomes derived from 3D cultures (3D EXO). Mesenchymal stem cells derived from human amnion (hAMSCs). Data are representative of three independent experiments.

EXO were isolated from hAMSC cultures at 90% confluence. The serum-free conditioned medium used was collected after 3 days of culture and centrifuged at 300 × g for 10 minutes to remove the debris. To further remove both cells and cell debris, the medium was centrifuged for 20 minutes at 16500 × g and then ultracentrifuged at 120000 × g for 90 minutes at 4°C to pellet the EXO. Total protein content of EXO preparations was determined using the Micro BCA Protein Assay Reagent Kit, following the manufacturer's specifications and using BSA (Thermo Scientific, USA) as a standard. Exosome purity was assessed by Western blot of markers of endoplasmic reticulum (Calnexin) and EXO (Alix) compartments (Figure 1(a)).

### 2.7. Nanoparticle Tracking Analysis (NTA)

Both size distribution and concentration of EXO were determined by NTA in a NanoSight NS3000 (Malvern Instruments Ltd., Malvern, UK). Samples were diluted 80 times with PBS to reach optimal concentration for instrument linearity, and the data were analyzed with NTA software version 3.1. (Build 3.1.54). Readings were taken on triplicate of 60 s at 25 frames per second, at a camera level set to 16, and with manual monitoring of temperature (Figures 1(b) and 1(c)).

### 2.8. Cell Migration Assay (xCELLigence)

Real-time monitoring of endothelial cell migration was done with the xCELLigence system (ACEA, USA) with the CIM-Plate 16. The upper chamber was seeded with 30000 HUVECs in DMEM serum-free medium. When cells migrated through the membrane into the bottom chamber in response to attractants (160 *μ*l of serum-free DMEM conditioned by each condition), they contacted and adhered to the electronic sensors, resulting in increased impedance. The cell index (CI) values reflecting impedance changes were automatically and continuously recorded every 15 minutes. Each culture condition was carried out in quadruplicate. Analyses were performed by RTCA Software 1.2 of the xCELLigence system.

### 2.9. Tube Formation Assay

Early-passage HUVECs were maintained in endothelial cell basal medium supplemented with BulletKit (EBM-2) on a culture flask coated with 0.1% gelatin and maintained at 37°C with 5% CO_2_ until 70% confluence. The tubulogenesis assay was performed in Matrigel™ (BD Biosciences). HUVECs were dispensed at 2 × 10^4^ cells/well (96-well microplates, Nunc, Wiesbaden, Germany) on top of the Matrigel™ in DMEM serum-free (control), DMEM serum-free with 5 *μ*g/ml EXO, or each conditioned medium (100 *μ*l). Following incubation at 37°C and 5% CO_2_ for 6 hours, the cells were visualized by microscopy using an EVOS™ FL Digital Inverted Fluorescence Microscope (Fisher Scientific, Scotland, UK). The number of nodes, the number of branching points, and the tube length were measured with ImageJ (US National Institutes of Health), analyzing approximately 15 fields per replicate (*n* = 3).

### 2.10. Induction of Osteogenic, Adipogenic, and Chondrogenic Differentiation

The differentiation assay was done in both 2D and 3D hAMSC cultures. The spheroid cells were dissociated into a single-cell suspension with 0.25% trypsin/EDTA and seeded to the culture plates for cell expansion. Osteogenic differentiation and adipogenic differentiation were evaluated by growing the cells for 14 days in *α* MEM with 10% FBS supplemented with osteogenic and adipogenic supplements, respectively (R&D Systems, USA). Medium with DMEM/F12 containing both ITS supplement (R&D Systems, USA) and chondrogenic supplement (R&D Systems, USA) was used for chondrogenic differentiation. A panel of antibodies consisting of anti-mFABP4, anti-hACAN, and anti-hOC was assayed by immunofluorescence to define the mature phenotypes of adipocytes, chondrocytes, and osteocytes, respectively (R&D Systems, USA). Samples were analyzed using an EVOS^™^ FL Digital Inverted Fluorescence Microscope (Fisher Scientific, Paisley, Scotland, UK). Signal intensities were calculated with ImageJ software.

### 2.11. Gene Expression Profile Analysis

We performed a molecular screening array based on TaqMan Array Cards (Life Technologies, USA), allowing analysis of selected genes related to both angiogenesis and inflammation. We also used TaqMan gene assay for analysis of EZH2 (Hs01016789_m1) and TERT (Hs00972650_m1). In addition, we performed real-time PCR using cDNA as the template in a 20 *μ*l reaction mixture containing SYBR Select Master Mix (Life Technologies, USA) and a specific primer pair for the following genes: *OCT4*, *SOX2*, *NANOG*, *PPARG*, *FABP4*, *LPL*, *OC*, *OPN*, *ALP*, *SOX9*, *ACAN*, *COL2A1*, and *GAPDH* (Table 1). Total RNA was extracted with the RNeasy Mini Kit and treated with DNAse (QIAGEN, Hilden, Germany). Subsequently, 100 ng of RNA was transcribed with the high-capacity RNA-to-cDNAkit protocol (Life Technologies, USA) in order to produce single-stranded cDNA. Samples were analyzed using the ABI Prism 7900HT Real-Time PCR System (Life Technologies, USA). The GAPDH gene was used as housekeeping gene. Furthermore, hierarchical cluster analysis of gene expression was used to group treatments with a similar expression pattern. Gene expression data of 34 genes assayed with TaqMan Array Cards were grouped using a hierarchical clustering algorithm in the Gene Cluster 3.0 program. A heat map was generated using Java TreeView program.

### 2.12. Profiling of Paracrine Factors

The levels of different paracrine factors involved in both angiogenesis and inflammation in each conditioned medium were determined using magnetic beads technology from Luminex™ with the ProcartaPlex Human Cytokine Chemokine Growth Factor (Affymetrix, Vienna, Austria), according to the manufacturer's instructions. In addition, the levels of TGF-*β* and PGE-2 were determined using the TGF-*β* Quantikine ELISA kit and the prostaglandin E2 parameter assay kit (R&D Systems, USA), respectively, following the manufacturer's instructions. Concentration of each factor was calculated from standard curves. Results from three independent experiments are shown as fold increase of each conditioned medium relative to HDFa-conditioned medium.

### 2.13. Anti-CD3/CD28 PBMC Stimulation Assay

Peripheral blood mononuclear cells (PBMCs) were obtained from heparinized whole blood samples or buffy coats from healthy subjects using density gradient centrifugation (Lymphoprep, Axis-Shield). To study the effect of both conditioned medium and EXO on CD3/CD28-stimulated PBMCs, the cells were seeded in 96-well plates (Corning, USA) at 1 × 10^5^ cells/well in serum-free DMEM (control) (200 *μ*l), DMEM serum-free with 5 *μ*g/ml EXO (194 *μ*l of DMEM, 2 *μ*l of each stimulus, and 2 *μ*l of EXO), or each conditioned medium (196 *μ*l of DMEM and 2 *μ*l of each stimulus). In order to evaluate cell proliferation, PBMCs were prelabeled with 0.5 *μ*M CellTrace™ CFSE Cell Proliferation Kit (Life Technologies, USA) according to the manufacturer's instructions and activated by 1 *μ*g/ml of anti-CD3 (clone UCHT1, 2 *μ*l) and 1 *μ*g/ml anti-CD28 (clone CD28.2, 2 *μ*l) monoclonal antibodies (BD Biosciences, USA). Cultures were carried out in triplicate in a final volume of 200 *μ*l, and cellular proliferation was assessed after 4 days. Nonadherent PBMCs were rescued from culture medium, washed in PBS, and analyzed with fluorescent-activated cell analysis using the FACS Aria II flow cytometer and FACS Diva software version 6.1.2 (BD Biosciences, Sunnyvale, CA, USA).

### 2.14. Statistics

All data are representative of at least triplicate in three independent experiments and are expressed as the mean ± standard deviation (SD). Data from different groups were compared using computerized statistical software with the ANOVA test. When ANOVA revealed *p* < 0.05, the data were further analyzed with Dunnet's *t*-tests. Differences were considered statistically significant at *p* < 0.05.

## 3. Results

### 3.1. Culture, Characterization, and Spheroid Formation of hAMSCs

**Figure 2:**
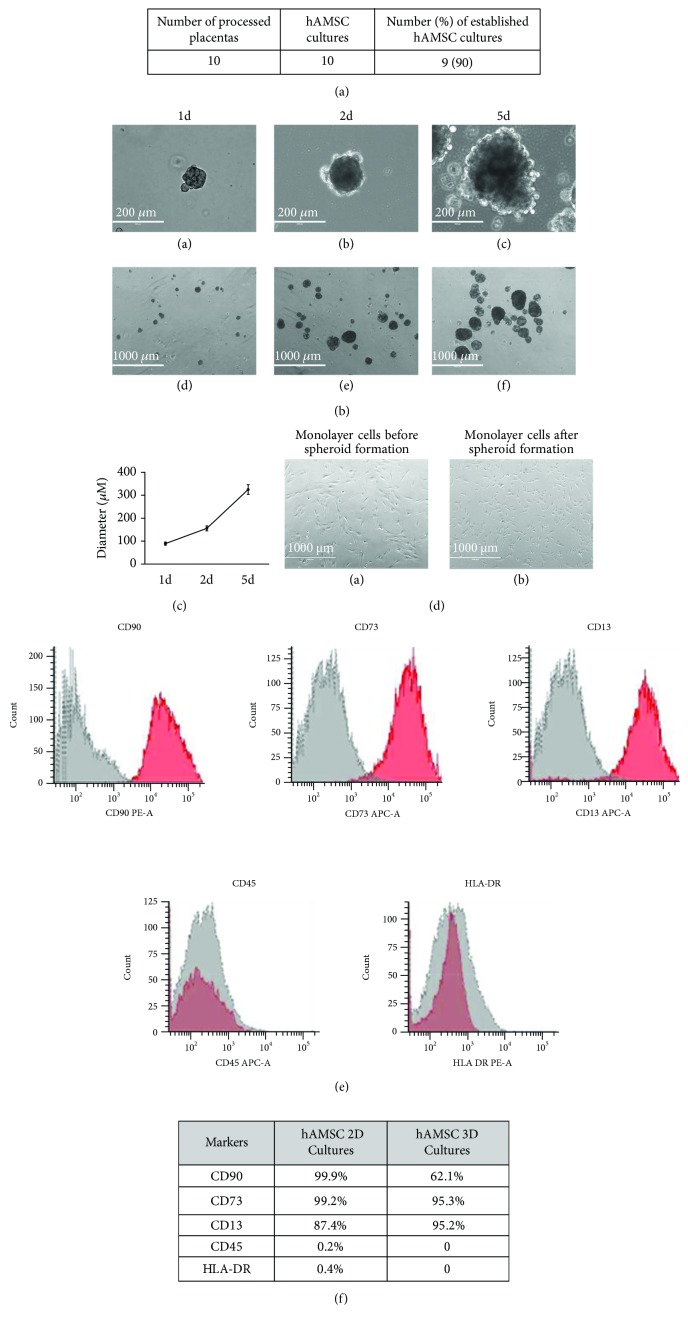
Human amnion mesenchymal stem cells (hAMSCs) grown as both monolayer and spheroids. (a) Number of established hAMSC cultures. (b) Representative images of hAMSC spheroids grown on low-binding plates at 1, 2, and 5 days after seeding. (c) The diameter of hAMSC spheroids after 1, 2, and 5 days of culture. (d) Monolayer cultures before spheroid formation and monolayer cultures after spheroid formation obtained from dissociated spheroids. (e) Representative images of FACS analysis of the surface marker in hAMSCs at passage 0. (f) FACS analysis of the surface marker in 2D hAMSC cultures and 3D hAMSC cultures at passage 2. Mesenchymal stem cells derived from human amnion-derived MSCs (hAMSCs); 1d: 1 day of culture; 2d: 2 days of culture; 5d: 5 days of culture. All data are expressed as means ± SD of triplicate in three independent experiments.

We established 90% of hAMSC culture from 10 processed placentas (Figure 2(a)), and from these cells grown in a suspended state (3D), we observed the generation of spheroids (Figure 2(b)). In this condition, cells spontaneously aggregated and formed compact multicellular spheroids 24 hours after suspension culture (Figure 2(b), A and D). Furthermore, we investigated the effects of culture time on the spheroid formation. After the spheroids had been allowed to aggregate for 24 hours, the spheroid diameter was quantified via bright-field microscopy (*n* = 10 per time point). The spheroid diameter increased with increasing culture time, with a diameter of 89 ± 6 *μ*m, 156 ± 10 *μ*m, and 325 ± 21 *μ*m after 1, 3, and 5 days, respectively (Figure 2(c)). Monolayer cultures before spheroid formation and monolayer cultures obtained from dissociated spheroids were compared morphologically (Figure 2(d)). It was found that monolayer cultures derived from spheroids were composed of small and elongated fibroblast-like cells. Moreover, common MSC surface markers in both hAMSC 2D cultures and hAMSC 3D cultures were assayed with flow cytometry. It was observed that the human MSC markers CD90, CD73, and CD13 were expressed in all cultures, whereas the cells were negative for CD45 and HLA-DR. At passage 0, the phenotype of hAMSCs was CD90 (84-99%), CD73 (66-99%), CD13 (75-89%), CD45 (0.2-2.9%), and HLA-DR (0.3-0.9%) (Figure 2(e)). However, hAMSC 2D cultures differed significantly from hAMSC 3D cultures in the expression level of CD90, which was lower in spheroids than in monolayer cultures (*p* < 0.05) (Figure 2(f)).

### 3.2. Paracrine Induction of Cell Migration and Tubulogenesis In Vitro Was Enhanced when hAMSCs Were Cultured as Spheroids

The functional effects of CM and EXO produced by both 2D and 3D cultures of hAMSCs were investigated concerning two essential aspects of the angiogenesis process: endothelial cell migration and the formation of endothelial cell capillary-like structures. We also included CM derived from both BM-MSCs and HDFa as control.

**Figure 3:**
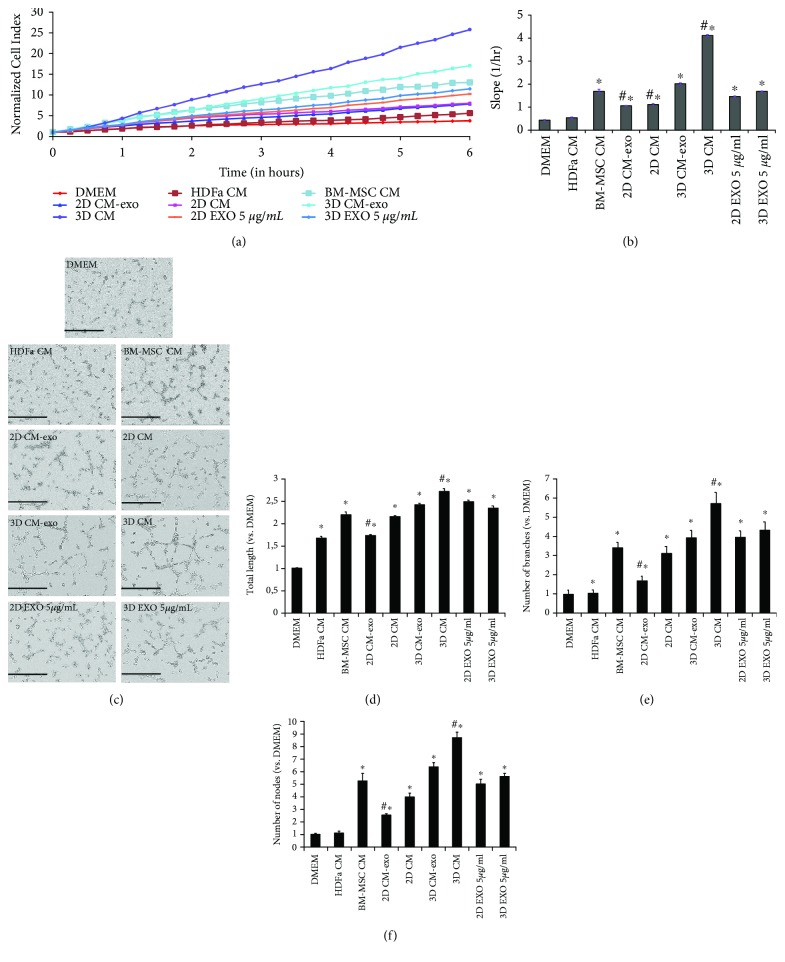
HUVEC migration assay and capillary-like formation assay. (a) Real-time migration monitoring of HUVECs with the xCELLigence system. (b) Slopes of migration curves. (c) Representative images of HUVECs on Matrigel, in contact with each conditioned medium. (d-f) Graphs represent a quantitative analysis of capillary length (d), branching point number (e), and node number (F). DMEM serum-free medium (DMEM). DMEM conditioned by HDFa (HDFa CM). DMEM conditioned by BM-MSCs (BM-MSC CM). Exosome-depleted DMEM conditioned by hAMSCs grown as monolayer (2D CM-exo). DMEM conditioned by hAMSCs grown as monolayer (2D CM). Exosome-depleted DMEM conditioned by hAMSCs grown as spheroids (3D CM-exo). DMEM conditioned by hAMSCs grown as spheroids (3D CM). 5 *μ*g/ml exosomes secreted by hAMSCs grown as monolayer (2D EXO 5 *μ*g/ml). 5 *μ*g/ml exosomes secreted by hAMSCs grown as spheroids (3D EXO 5 *μ*g/ml). Data are means ± SD of quadruplicate in three independent experiments. ^∗^*p* < 0.05 vs. DMEM and #*p* < 0.05 vs. CM BM-MSCs. Bar = 400 *μ*m.

We observed a noticeable increase in HUVEC migration in the presence of 3D CM of approximately 4-, 2-, and 8-fold when compared with 2D CM (with or without EXO), BM-MSC CM, and HDFa CM or DMEM, respectively. Furthermore, 3D CM showed a significant increase in HUVEC migration (2-fold) compared with same medium without EXO. Interestingly, both 2D and 3D EXO at 5 *μ*g/ml concentration induced a 4-fold increase of HUVEC migration compared with control DMEM (Figures 3(a) and 3(b)).

We further addressed whether CM and EXO produced by both 2D and 3D cultures of hAMSCs would induce the *in vitro* formation of capillary-like structures by endothelial cells and a similar picture was observed. In particular, tubule formation by HUVECs consistently showed a significant increase in the number of nodes, the number of branches, and the length in the presence of 3D CM compared with 3D CM-exo, 2D CM (with or without EXO), BM-MSC CM, and HDFa CM, respectively. Furthermore, EXO were also able to induce *in vitro* formation of capillary-like structures by endothelial cells. Both 2D and 3D EXO at 5 *μ*g/ml concentration led to an increase in formation of capillary-like structures compared with control DMEM. Interestingly, significant differences in tubule formation were observed in both 2D and 3D CM with or without EXO, with a greater effect of CM compared with CM-exo (Figures 3(c)–3(f)).

### 3.3. Paracrine Inhibition of PBMC Proliferation by hAMSCs

**Figure 4:**
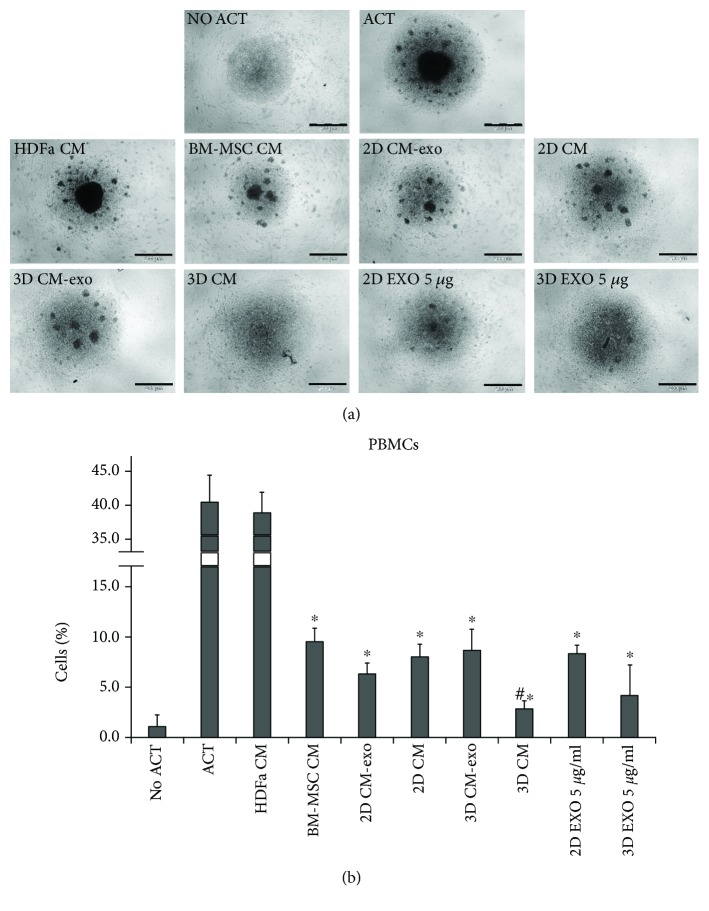
Inhibition of activated PBMCs. (a) Representative images of unstimulated (No ACT) and CD3/CD28-stimulated (ACT) PBMCs grown in each conditioned medium. (b) FACS analysis of PBMC after 4 days of culture in each conditioned medium. DMEM without CD3/CD28 (No ACT). DMEM with CD3/CD28 (ACT). DMEM conditioned by HDFa (HDFa CM). DMEM conditioned by BM-MSCs (BM-MSC CM). Exosome-depleted DMEM conditioned by hAMSCs grown as monolayer (2D CM-exo). DMEM conditioned by hAMSCs grown as monolayer (2D CM). Exosome-depleted DMEM conditioned by hAMSCs grown as spheroids (3D CM-exo). DMEM conditioned by hAMSCs grown as spheroids (3D CM). 5 *μ*g/ml exosomes secreted by hAMSCs grown as monolayer (2D EXO 5 *μ*g/ml). 5 *μ*g/ml exosomes secreted by hAMSCs grown as spheroids (3D EXO 5 *μ*g/ml). Data are means ± SD of triplicate in three independent experiments. ^∗^*p* < 0.05 vs. ACT and #*p* < 0.05 vs. CM BM-MSCs. Bar = 200 *μ*m.

We tested the effects of CM and EXO produced by both 2D and 3D cultures of hAMSCs on PBMC proliferation stimulated with both anti-CD3 and anti-CD28. The inhibitory effect of hAMSC-conditioned medium was time dependent. We found that conditioned media derived from both 1 day and 2 days of cultures did not inhibit proliferation of activated PBMCs (data not shown) but it was inhibited by 3 days of CM derived from either BM-MSCs or hAMSCs. In particular, there was a significant reduction in PBMC proliferation when these cells were grown in BM-MSC CM (76% inhibition vs. ACT treatment; *p* value: 0.0002). We also observed an evident inhibition in PBMC proliferation with both 2D CM-exo and 2D CM (84% and 80% inhibition, respectively, vs. ACT treatment; *p* value: 0.0001 and 0.0002, respectively), with no significant differences between them. On the other hand, 3D CM (that showed an inhibition of 93% vs. ACT treatment; *p* value: 0.00009) showed a significant increase in inhibiting PBMC proliferation compared with both the same medium without EXO (that showed an inhibition of 78% vs. ACT treatment; *p* value: 0.0003) and BM-MSC CM, with a *p* value of 0.01 and 0.002, respectively. 2D and 3D EXO at 5 *μ*g/ml concentration inhibited PBMC proliferation (79% and 89% inhibition, respectively, vs. ACT treatment; *p* value: 0.0002 for both) with no significant differences between them (Figure 4).

### 3.4. Spheroid Formation of hAMSCs Increased the Expression of Both Angiogenic Growth Factors and Immunosuppressive Factors

**Figure 5:**
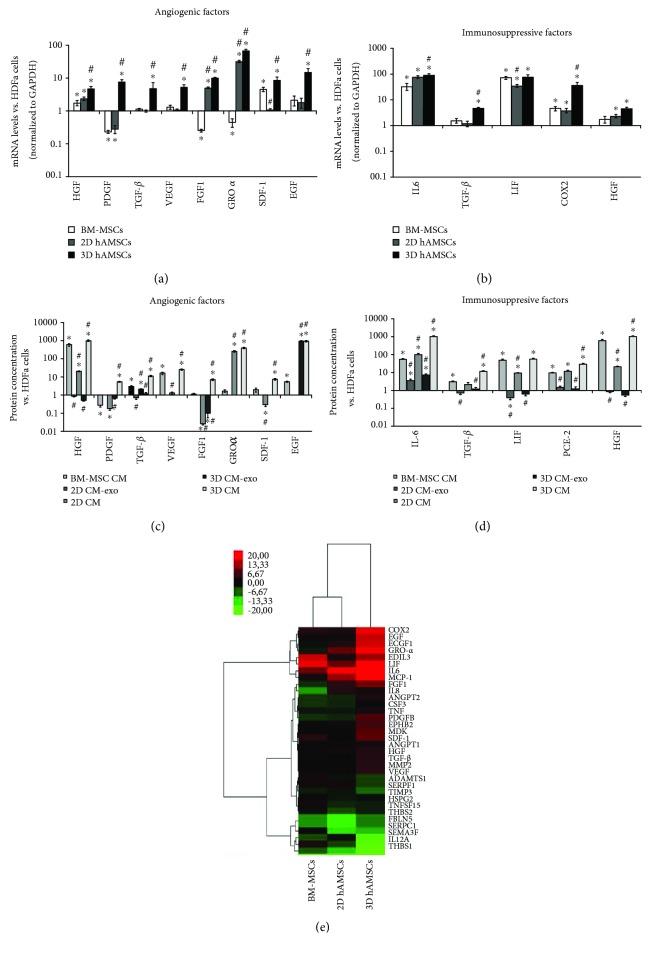
Expression analysis of angiogenic and immunosuppressive factors. Both gene (a, b) expression and protein (c, d) expression were assayed after 3 days of cultures in cells and CM, respectively. (a) Gene expression of angiogenic factor. (b) Gene expression of immunosuppressive factor. (c) Protein expression of angiogenic factor. (d) Protein expression of immunosuppressive factor. (e) Hierarchical clustering of gene expression profile. Transcript levels were normalized to those of GAPDH and expressed as fold change vs. gene expression values of HDFa. Bone marrow mesenchymal stem cells (BM-MSCs). Amnion mesenchymal stem cells grown in two-dimensional cultures (2D hAMSCs). Amnion mesenchymal stem cells grown in three-dimensional cultures (3D hAMSCs). DMEM conditioned by BM-MSCs (BM-MSC CM). Exosome-depleted DMEM conditioned by hAMSCs grown as monolayer (2D CM-exo). DMEM conditioned by hAMSCs grown as monolayer (2D CM). Exosome-depleted DMEM conditioned by hAMSCs grown as spheroids (3D CM-exo). DMEM conditioned by hAMSCs grown as spheroids (3D CM). Data are means ± SD of triplicate in three independent experiments. ^∗^*p* < 0.05 vs. HDFa and #*p* < 0.05 vs. BM-MSCs.

The gene expression of both angiogenic growth factors (*HGF*, *PDGF*, *TGF-β*, *VEGF*, *FGF1*, *GRO-α*, *SDF-1*, and *EGF*) and immunosuppressive factors (*IL6*, *TGF-β*, *LIF*, *COX2*, and *HGF*) was analyzed with real-time PCR. The angiogenic genes were significantly upregulated in 3D hAMSCs when compared with both BM-MSCs and 2D hAMSCs (Figure 5(a)). Moreover, the immunosuppressive factors, except LIF, were also significantly upregulated in 3D hAMSCs compared with BM-MSCs, whereas all the factors tested, except IL6, were significantly upregulated in 3D hAMSCs compared with 2D hAMSCs (Figure 5(b)).

Protein analysis further confirmed the gene expression data (Figures 5(c) and 5(d)). Remarkable differences in protein expression were observed in both 2D hAMSCs and 3D hAMSCs between the medium with and the medium without EXO, with a greater expression of both angiogenic growth factors and immunosuppressive factors in CM compared with CM-exo, except EGF.

The genes were also grouped using a hierarchical cluster analysis, which showed a similar pattern of expression between 2D hAMSCs and BM-MSCs; instead, a different pattern of expression was found for 3D hAMSCs (Figure 5(e)).

### 3.5. hAMSCs Cultured as Spheroids Increased Both the Expression of Pluripotent Markers and the Mesenchymal Stromal Cell Differentiation Potential

In order to examine whether spheroid culture maintained or increased the multipotency and the differentiation capacity of hAMSCs, cell spheroids were dissociated in a single-cell suspension, plated onto culture flasks, grown as a monolayer, and used for further analysis. Plated cells retained the ability to adhere and proliferate on a plastic surface with low mortality (data not shown).

**Figure 6:**
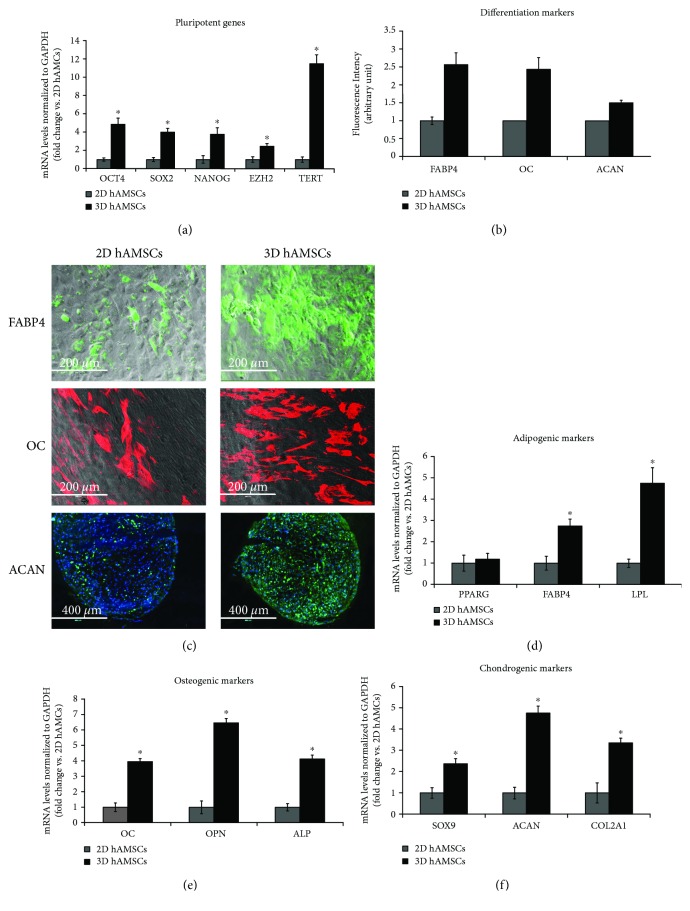
Gene expression and immunofluorescence analysis of adipogenic, osteogenic, and chondrogenic markers and gene expression analysis of pluripotency markers. (a) Gene expression of selected pluripotent genes. (b) Graphics depict FABP4, OC, and ACAN fluorescence intensity in hAMSCs grown as both monolayer and spheroids. (c) Immunofluorescence staining localization of FABP4, OC, and ACAN in hAMSCs grown as both monolayer and spheroids. (d) Gene expression of selected adipogenic markers. (e) Gene expression of selected osteogenic markers. (f) Gene expression of selected chondrogenic markers. Transcript levels were normalized to those of GAPDH and expressed as fold change vs. gene expression values of 2D hAMSCs. Amnion mesenchymal stem cells grown in two-dimensional cultures (2D hAMSCs). Amnion mesenchymal stem cells grown in three-dimensional cultures (3D hAMSCs). Data are means ± SD of triplicate in three independent experiments. ^∗^*p* < 0.05 vs. 2D hAMSCs.

First, we evaluated the expression of pluripotency-associated transcription factors (*OCT4*, *SOX2*, *NANOG*, *EZH2*, and *TERT*) by real-time PCR. Notably, spheroid-derived hAMSCs exhibited significantly greater expression levels of *OCT4*, *SOX2*, *NANOG*, *EZH2*, and *TERT* with 5-, 4-, 4-, 2-, and 11-fold increases, respectively, compared with monolayer hAMSCs (Figure 6(a)). Cell multipotency was then assessed by the ability of hAMSCs to differentiate *in vitro* into adipocyte-, osteoblast-, and chondrocyte-like cells. Adipogenic differentiation, osteogenic differentiation, and chondrogenic differentiation were detected by both immunofluorescence assay of *FABP4*, *OC*, and *ACAN*, respectively, and gene expression analysis of adipogenic (*PPARG*, *FABP4*, and *LPL*), osteogenic (*OC*, *OPN*, and *ALP*), and chondrogenic (*SOX9*, *ACAN*, and *COL2A1*) markers. As expected, all three differentiation processes were obtained with hAMSCs, but were significantly enhanced by cell spheroids, as confirmed by both protein analysis and gene expression analysis of differentiation markers. Indeed, cells obtained from three-dimensional cultures and plated back under two-dimensional conditions showed a greater ability to differentiate than cells grown in conventional two-dimensional cultures at similar passages. In particular, immunofluorescence assay shows an upregulation of *FABP4* (2.6-fold), *OC* (2.4-fold), and *ACAN* (1.5-fold) proteins (Figures 6(b) and 6(c)) and gene expression analysis shows a significant upregulation of adipocyte-specific markers, *FABP4* (2.7-fold) and *LPL* (4.7-fold) (Figure 6(d)), osteocyte-specific markers, *OC* (3.9-fold), *OPN* (6.4-fold), and *ALP* (4.1-fold) (Figure 6(e)), and chondrocyte-specific markers, *SOX9* (2.4-fold), *ACAN* (4.7-fold), and *COL2A1* (3.3-fold) (Figure 6(f)).

## 4. Discussion

Increasing evidence shows that MSCs play a role in tissue repair and regeneration, with the secretion of soluble factors that enhance the response of damaged tissues through paracrine regulation of local cells [3, 19–23]. Paracrine secretion by MSCs was first identified by Haynesworth et al. [45]. They reported that MSCs produce and release a broad range of growth factors, chemokines, and cytokines that modulate the action of adjacent cells. Indeed, these secreted factors increase angiogenesis, reduce apoptosis and fibrosis, stimulate extracellular matrix remodeling, and regulate immune responses. Therefore, MSCs through paracrine secretion induce regeneration for rescuing injured cells, decreasing tissue injury, and finally accelerating organ repair [20]. Several studies have investigated the therapeutic effects of MSC-derived paracrine factors on different disorders, including immune diseases, neurological diseases, liver injury, acute kidney failure, and cardiovascular diseases. These studies have indicated that molecules secreted by MSCs perform an effective role as mediators that either directly activate the target cells or stimulate neighboring cells to secrete active factors [20, 46]. Recently, it has been documented that MSCs also release numerous extracellular vesicles that participate in tissue regeneration by transferring information to damaged cells or tissue and exert biological activity similar to the MSCs [24, 26, 28, 46, 47].

Many studies investigating the regenerative properties of MSCs were conducted using MSCs grown in monolayer cultures [48], while Cheng et al. found an enhancement in wound healing rates after treatment with spheroid-derived adipose stem cells using a number of cells three times lower with respect to other studies [49]. Recently, several publications have suggested that MSC 3D cultures may be more appropriate than traditional 2D systems for increasing the therapeutic potential of these cells [10, 35, 36], as well as increasing the expression of angiogenic and/or anti-inflammatory factors [37, 38]. Moreover, cell spheroids do not undergo the influence of substrate attachments, which normally induce cellular senescence and the lowering of the differentiation potential, thus providing the favorable conditions for stem cell growth [36, 50, 51]. The molecular processes that increase the expression of pluripotent markers, angiogenic and immunomodulatory factors in spheroid-derived MSCs, are unclear. One evident change upon spheroid formation is the development of a hypoxic environment in the core of each spheroid [52]. Oxygen reaches the interior of spheroids through diffusion, which makes the internal core of spheroids hypoxic [51]. Indeed, hypoxia-associated genes such as VEGF, PDGF, TGF-*β* HGF, SDF-1, EGF, IL6, LIF, and COX2 are overexpressed among the upregulated genes in MSC spheroids, where hypoxia-inducible factor (HIF) is a master transcription factor that regulates the expression of these angiogenic and immunomodulatory genes [53–55]. Also, the alteration in mechanophysical properties might be such a significant event in MSC spheroids [51]. Guo et al. showed that hAMSC growth in 3D culture acquires epigenetic changes, increasing their clonogenicity and differentiation potency and, as a result, the expression levels of genes involved in stem cell potency were changed [39]. Thus, epigenetic regulation appears to be one of the underlying molecular mechanisms causing the drastic change in the gene expression profile in MSC spheroids.

In our study, we aimed to (1) obtain MSCs from the human placenta amniotic membrane, (2) establish a procedure for obtaining MSC spheroids with a secretome that shows improved angiogenetic and immunosuppressive capacity compared with that obtained from the same cells grown as monolayer cultures, and (3) examine and test *in vitro* both CM and EXO derived from 3D MSCs to study their paracrine therapeutic potential in both angiogenic and inflammatory pathways.

We showed that cell spheroids were successfully cultured and maintained in suspension for at least 5 days and this culture system promoted maintenance of stemness as observed in greater expression of key stemness markers Oct4, SOX2, NANOG, EZH2, and TERT. Furthermore, the results of the osteogenic, adipogenic, and chondrogenic inductions suggest again that the spheroids possess strong multipotency and stemness. We also analyzed a representative group of factors involved in key pathways of the tissue regeneration process, including angiogenesis pathways and immunomodulatory pathways. Our results show that both angiogenic and immunosuppressive factors are consistently produced and secreted at higher levels by hAMSC spheroids compared with monolayer cultures. Indeed, the protein production of HGF, PDGF, TGF-*β*, VEGF, FGF1, GRO-*α*, SDF-1, and EGF, primarily involved in the angiogenesis pathways, was obtained at much higher levels in 3D CM compared with 2D CM. These growth factors are very important in tissue regeneration because they affect tissue repair, play an essential role in promoting angiogenesis, and can induce cell proliferation, migration, and the secretion of angiopoietic factors during angiogenic process [56, 57]. Our data suggest an increased paracrine potential of 3D CM to induce angiogenesis, as also confirmed by our *in vitro* tubulogenesis and endothelia cell migration results. Furthermore, our study found that both 2D and 3D hAMSC-derived EXO at 5 *μ*g/ml concentration can induce capillary-like formation and endothelial cell migration similarly to BM-MSC CM and without significant differences between 2D and 3D EXO. In our experimental conditions, different key factors of the immunosuppression process were also overproduced in 3D hAMSCs compared with 2D hAMSCs. MSCs exert their immunomodulatory effects through multiple mechanisms also mediated by IL6, LIF, and PGE2, which induce suppression of T-cell proliferation [9, 33]. Furthermore, HGF and TGF-*β* also possess immunomodulatory activity. Indeed, the treatment of dendritic cells with HGF reduces the ability to induce activation of Th1 cells [58] and TGF-*β* contributes to suppress activation of T lymphocyte proliferation [59]. Our data are consonant with these observations. We found an upregulation of IL6, LIF, PGE2, HGF, and TGF-*β* in 3D CM compared with 2D CM, suggesting an increased paracrine potential of 3D CM to induce immunosuppression, as confirmed by our *in vitro* assay of both 2D and 3D CM to inhibit activation of PBMC proliferation. Moreover, our study found that both 2D and 3D hAMSC-derived EXO treatments significantly suppressed the proliferation of activated PBMCs at 5 *μ*g/ml without significant differences between 2D and 3D EXO.

It has been shown that TGF-*β* is detected in microvesicles derived from MSCs [60]. We found profound differences in the protein expression between complete conditioned medium and exosome-depleted conditioned medium. Indeed, we found that all angiogenic and immunosuppressive factors tested, except for TGF-*β* in 2D CM and EGF in 3D CM, were expressed at higher levels only in conditioned medium that included EXO. This is in line with *in vitro* studies that show a greater effect of both 2D and 3D CM compared with exosome-depleted conditioned medium, except for 2D CM inhibition of PBMC proliferation, probably due to the lack of significant differences in the TGF-*β* content observed between 2D CM and 2D CM-exo. These results revealed that both angiogenic and immunosuppressive effects of hAMSC CM were, at least partly, attributable to EXO.

As previously documented by both *in vitro* and *in vivo* studies that cells isolated from the mesenchymal region of the human amniotic membrane possess both immunoregulatory and angiogenesis properties [21, 23], our results further confirm that the conditioned medium of hAMSCs promoted, *in vitro*, angiogenesis and immunosuppression. We found that these effects were enhanced when the cells were grown in scaffold-free three-dimensional cultures. Furthermore, hAMSC-derived EXO also showed both angiogenic and immunosuppressive effects with lower capacity compared with 3D CM. These findings are consistent with previous reports [61]. Moreover, we found that the effects of both 2D and 3D CM were reduced after depleting EXO in the CM, which is consistent with previous reports [61]. We also compared the effects of CM and their EXO derived from hAMSCs with CM derived from BM-MSCs, a population of MSCs well studied for its potential use in regenerative medicine. In our experimental conditions, 3D CM of hAMSCs showed a greater paracrine effect compared with CM of BM-MSCs, while similar paracrine effects were observed between BM-MSC CM and both 2D and 3D hAMSC-derived exosomes.

In conclusion, our study confirms that the placenta could be considered an excellent source of MSCs and our 3D culture system represents a reproducible and scalable system for the maintenance of MSC spheroids and for the production of both CM and EXO that can be considered promising for cell-free therapies usable in the field of regenerative medicine.

## Figures and Tables

**Table 1:** List of primers used for quantitative PCR.

Gene		Primer sequence (5′-3′)	GenBank accession ID	Amplicon length
OCT4	Forward	5′-TCGAGAAGGATGTGGTCCGA-3′	NM_002701.5	93
	Reverse	5′-GCCTCAAAATCCTCTCGTTG-3′		
SOX2	Forward	5′-TGGCGAACCATCTCTGTGGT-3′	NM_003106.3	110
	Reverse	5′-CCAACGGTGTCAACCTGCAT-3′		
NANOG	Forward	5′-CCTGTGATTTGTGGGCCTG-3′	NM_001297698.1	77
	Reverse	5′-GACAGTCTCCGTGTGAGGCAT-3′		
PPARG	Forward	5′-AGCCTCATGAAGAGCCTTCCAAC-3′	NM_138712.3	121
	Reverse	5′-TCTCCGGAAGAAACCCTTGCATC-3′		
FABP4	Forward	5′-AAAGTCAAGAGCACCATAACC-3′	NM_001442.2	199
	Reverse	5′-TTCAATGCGAACTTCAGTCC-3′		
LPL	Forward	5′-TCATTCCCGGAGTAGCAGAGT-3′	NM_000237.2	124
	Reverse	5′-GGCCACAAGTTTTGGCACC-3′		
OC	Forward	5′-TAGTGAAGAGACCCAGGCGCTA-3′	NM_199173.5	108
	Reverse	5′-TCACAGTCCGGATTGAGCTCA-3′		
OPN	Forward	5′-TTGCAGCCTTCTCAGCCAA-3′	NM_001040058.1	75
	Reverse	5′-GGAGGCAAAAGCAAATCACTG-3′		
ALP	Forward	5′-ACTGGTACTCAGACAACGAGAT-3′	NM_000478.5	96
	Reverse	5′-ACGTCAATGTCCCTGATGTTATG-3′		
SOX9	Forward	5′-GGAAGTCGGTGAAGAACGGG-3′	NM_000346.3	320
	Reverse	5′-TGTTGGAGATGACGTCGCTG-3′		
ACAN	Forward	5′-ACTTCCGCTGGTCAGATGGA-3′	NM_001135.3	110
	Reverse	5′-TCTCGTGCCAGATCATCACC-3′		
COL2A1	Forward	5′-GAGACAGCATGACGCCGAG-3′	NM_001844.4	66
	Reverse	5′-GCGGATGCTCTCAATCTGGT-3′		
GAPDH	Forward	5′-GCATCTTCTTTTGCGTCG-3′	NM_002046.5	180
	Reverse	5′-TGTAAACCATGTAGTTGAGGT-3′		

## Data Availability

The data used to support the findings of this study are included within the article. The GenBank accession ID for each primer used for RT-PCR data of this study is included in the article in Table 1. Other data used to support the results of this study are available from the corresponding author upon request.
